# Acute visual loss as the first ocular symptom in a Sjögren’s syndrome patient with bilateral common carotid artery occlusion: a case report

**DOI:** 10.1186/s12886-021-02177-x

**Published:** 2021-11-27

**Authors:** Yi Wan, Hung-Chi Chen, Chia-Yi Lee, Hung-Yu Lin, Chan-Wei Nien

**Affiliations:** 1 Department of Ophthalmology, Show Chwan Memorial Hospital, Changhua, Taiwan; 2 Department of Ophthalmology, Chang Gung Memorial Hospital, Linkou, Taiwan; 3 Department of Medicine, Chang Gung University College of Medicine, Taoyuan, Taiwan; 4 Center for Tissue Engineering, Chang Gung Memorial Hospital, Linkou, Taiwan; 5 Institute of Medicine, Chung Shan Medical University, Taichung, Taiwan; 6 Department of Exercise and Health Promotion, Chung Chou University of Science and Technology, Changhua, Taiwan; 7 Department of Optometry, Chung Shan Medical University, Taichung, Taiwan; 8 Department of Optometry, Central Taiwan University of Science and Technology, Taichung, Taiwan

**Keywords:** Sjögren’s syndrome, Common carotid artery occlusion, Bilateral common carotid artery occlusion, Ocular ischemic syndrome, Acute visual loss

## Abstract

**Background:**

Sjögren’s syndrome may be a risk factor for carotid artery stenosis. Bilateral common carotid artery occlusion (BCCAO) in a patient with Sjögren’s syndrome was not reported before. In this report, we describe a female with Sjögren’s syndrome who had acute visual loss due to ocular ischemic syndrome (OIS) with BCCAO.

**Case presentation:**

A 50-year-old female with Sjögren’s syndrome visited our clinic with acute visual loss in the left eye. The best corrected visual acuity (BCVA) was 2/100 in the left eye, and the intraocular pressure (IOP) was normal in both eyes. Ocular ischemic change was observed during the ophthalmic examination. Aortography and computed tomography angiography (CTA) showed nearly total occlusion of the bilateral CCA. Thus, OIS with BCCAO was diagnosed. The vision in the left eye improved to 30/100 after carotid artery stenting for the left common carotid artery.

**Conclusions:**

BCCAO may be present in patients with Sjögren’s syndrome. Large vessel abnormalities should be considered when acute visual loss is found in a patient with Sjögren’s syndrome.

## Background

Sjögren’s syndrome can induce visual dysfunction, more commonly due to ocular surface disease and other inflammatory conditions [1]. Though atherosclerotic and nonatherosclerotic cardiovascular disease is a less often discussed topic in Sjögren’s syndrome [2, 3], occlusions of major cerebral arteries in a Sjögren’s patient has been reported [4]. However, there was no ophthalmic presentation in that case report.

Common carotid artery occlusion (CCAO) is a rare condition and is diagnosed in about 3% of symptomatic patients with cerebrovascular disease [5]. The risk factors of CCAO include hypertension (HTN), a history of smoking, and diabetes mellitus (DM) [6, 7]. The blood supply to the eye stems from the common carotid artery, thus, a CCAO can present with ocular ischemic syndrome (OIS) [8]. The most common clinical features of OIS include acute visual loss, ischemic ocular pain, and various anterior and posterior segment signs [8].

There has been only two cases of CCAO with the neuro-ophthalmic presentation reported; however, detailed imaging was not present [9]. Up to now, there is no documents of bilateral CCAO (BCCAO) cases in a patient with Sjögren’s syndrome, HTN, and DM. Therefore, we report a rare case of bilateral common carotid artery occlusion in a patient with Sjögren’s syndrome and complex pathogenesis using various imaging modalities.

## Case presentation

A 50-year-old female visited our clinic with sudden visual loss in her left eye.

The past medical history included retinal vasculitis of the right eye, Sjogren syndrome, coronary heart disease, type 2 DM, and HTN. The patient had DM and HTN for about 3 years. Both DM and HTN are well controlled in the past few years. This patient suffered from right tinnitus, vertigo, persistent vomiting, and dry mouth 2 years ago. Serological examination and labial gland biopsy revealed Sjögren’s syndrome. The patient started immunosuppressive drugs with prednisolone 5-10 mg/day and methotrexate 10 mg/week since then. The rheumatologist tapered prednisolone from 5 to 10 mg/day to 2.5 mg/day and kept methotrexate 10 mg/week 3 months before this event. Before this event, the best corrected visual acuity (BCVA) was counting fingers (CF)/20 cm in the right eye and 20/25 in the left eye. Recently, she experienced dizziness and walking instability. Sudden decrease in vision was noted when she squatted.

**Fig. 1:**
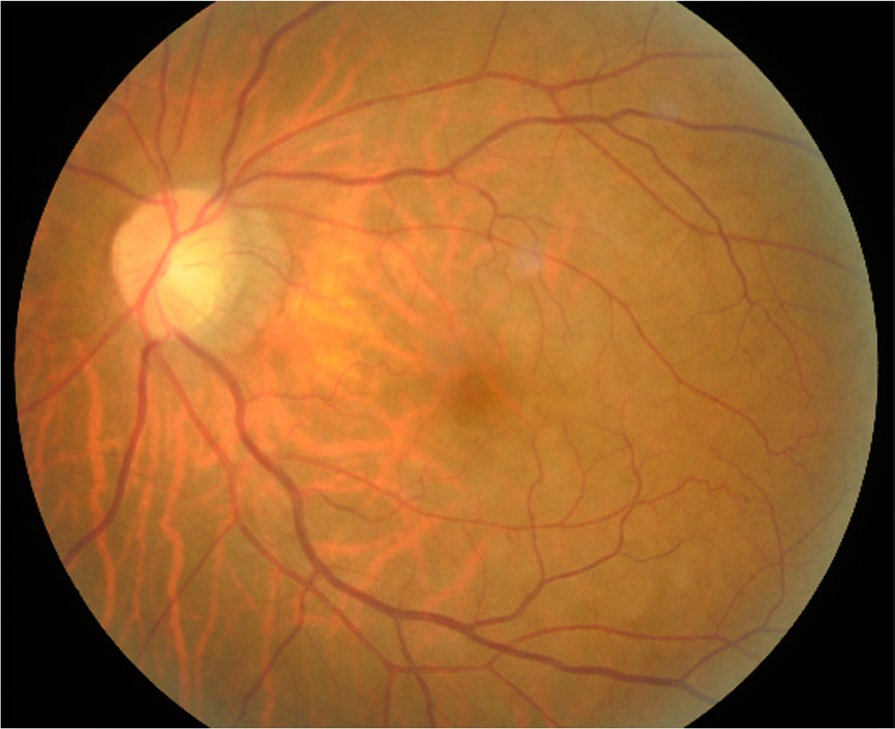
Fundus photograph of the left eye. Fundus photograph of the left eye showed venous dilation and attenuated arterioles

**Fig. 2:**
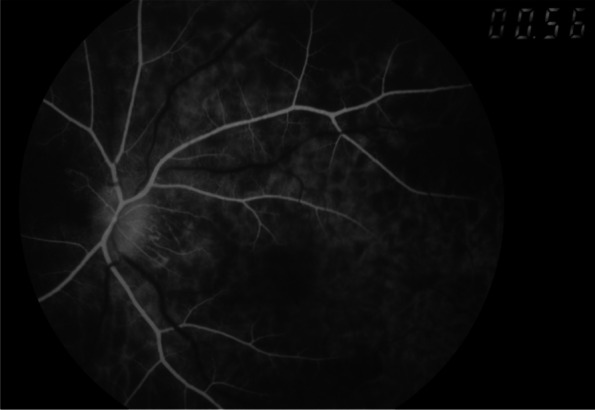
FAG image of the left eye. FAG image taken at 56 s (arterial phase) demonstrated prolonged arm-to-retina circulation time and a patchy choroidal filling pattern

**Fig. 3:**
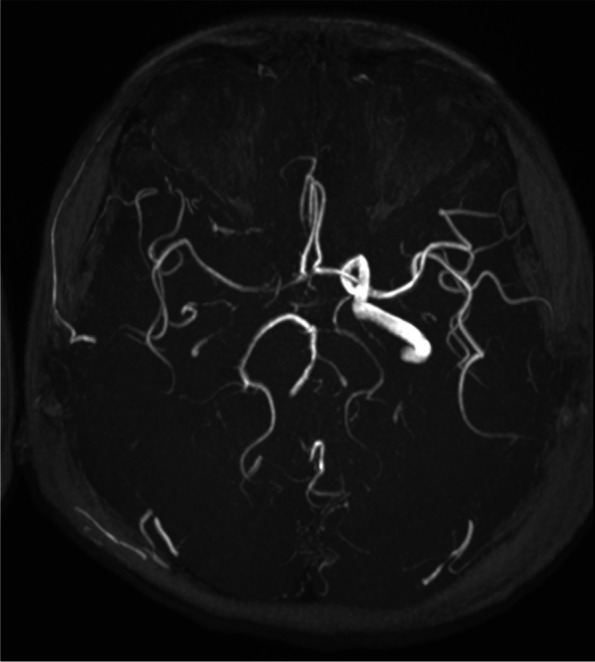
Brain MRA. MRA showed a total absence of right internal and external carotid arteries as well as left extracranial carotid arteries

**Fig. 4:**
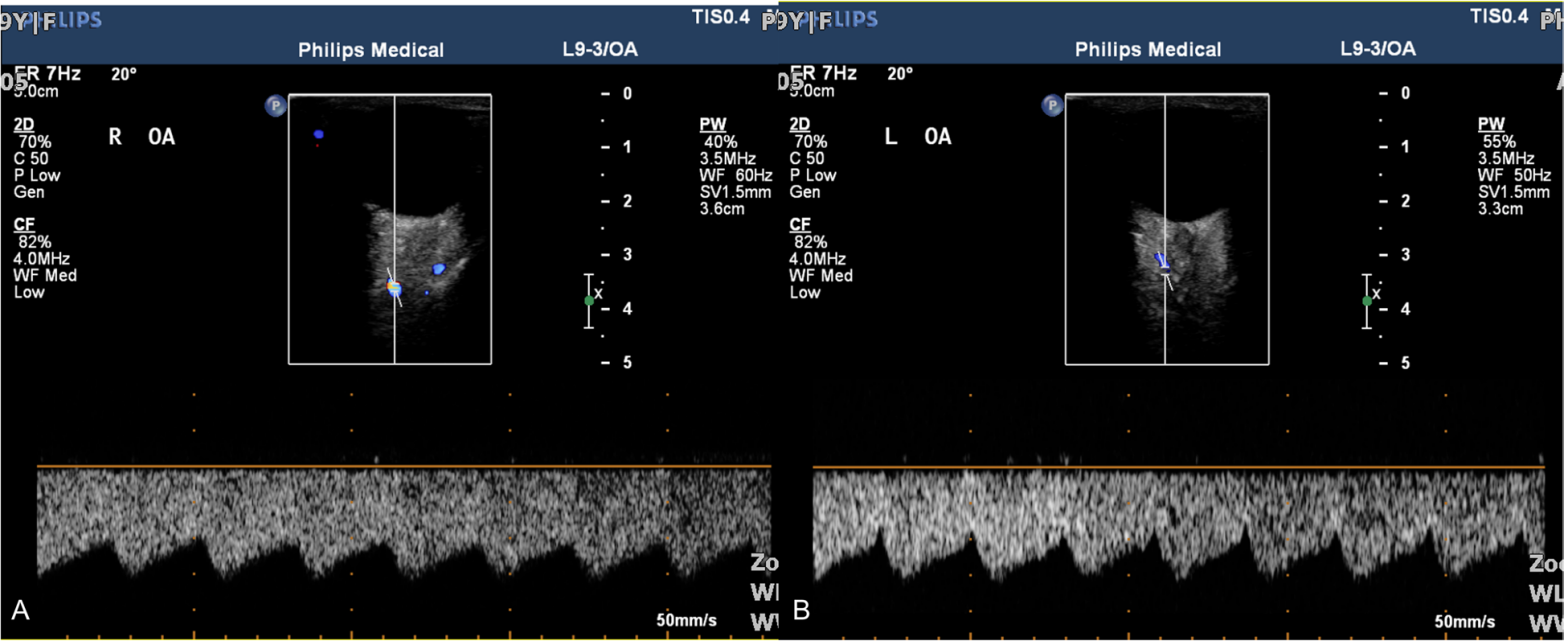
Ultrasonography of the ophthalmic artery. Ultrasonography of the ophthalmic artery showed reverse blood flow in the bilateral ophthalmic arteries

**Fig. 5:**
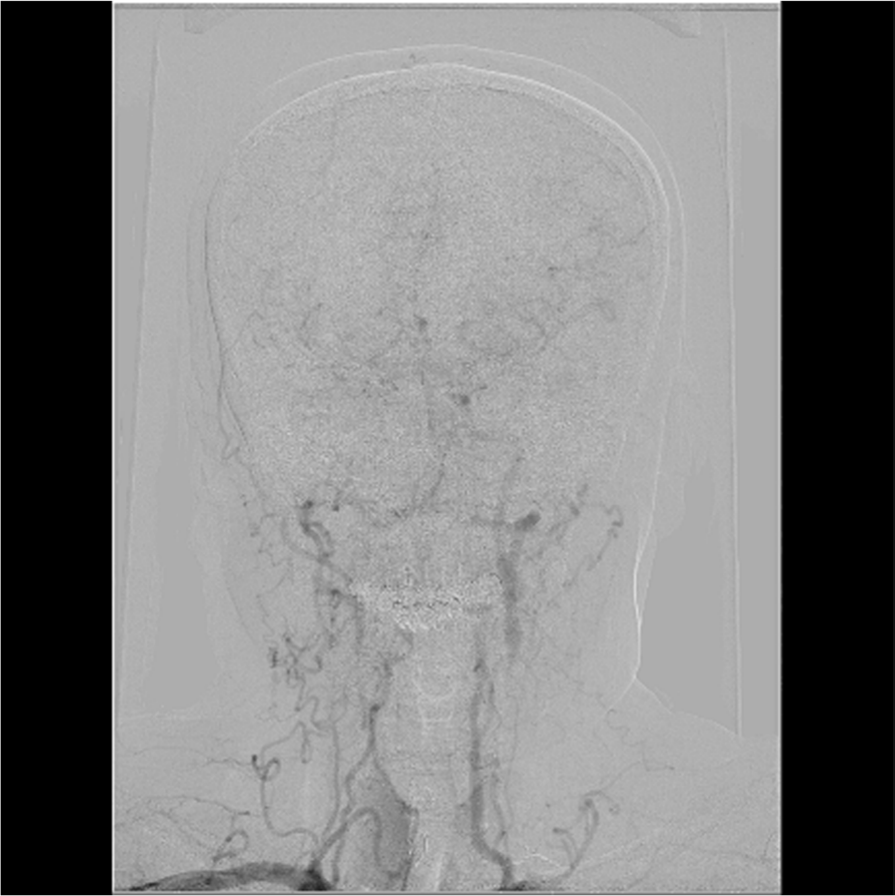
Aortography. Aortography showed total occlusion of the bilateral common carotid artery

**Fig. 6:**
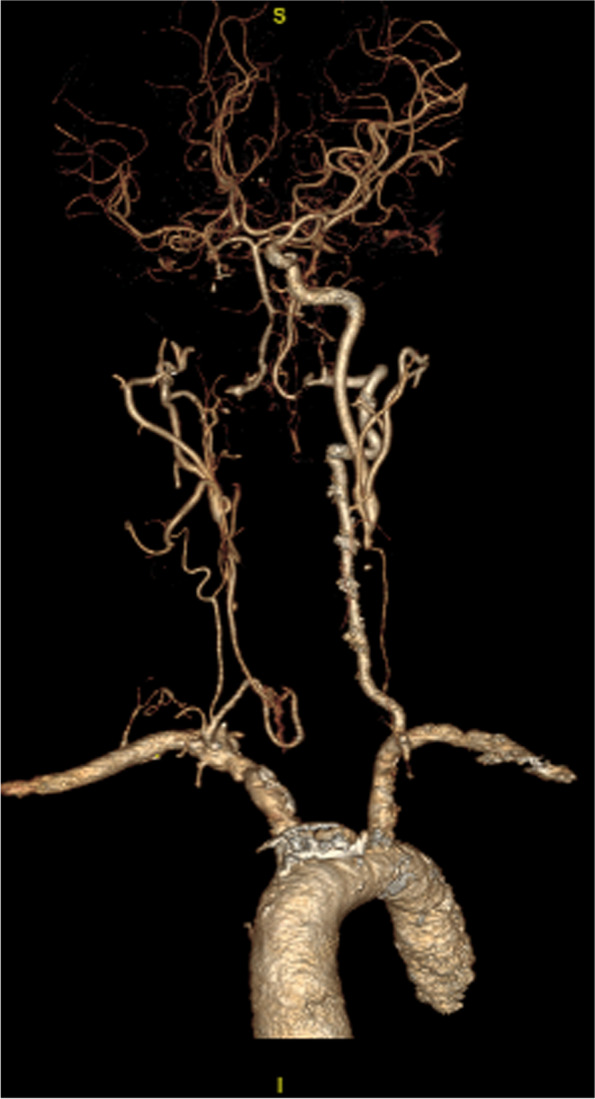
CTA. CTA showed nearly total occlusion of the bilateral CCA and nearly total occlusion of right ICA

**Fig. 7:**
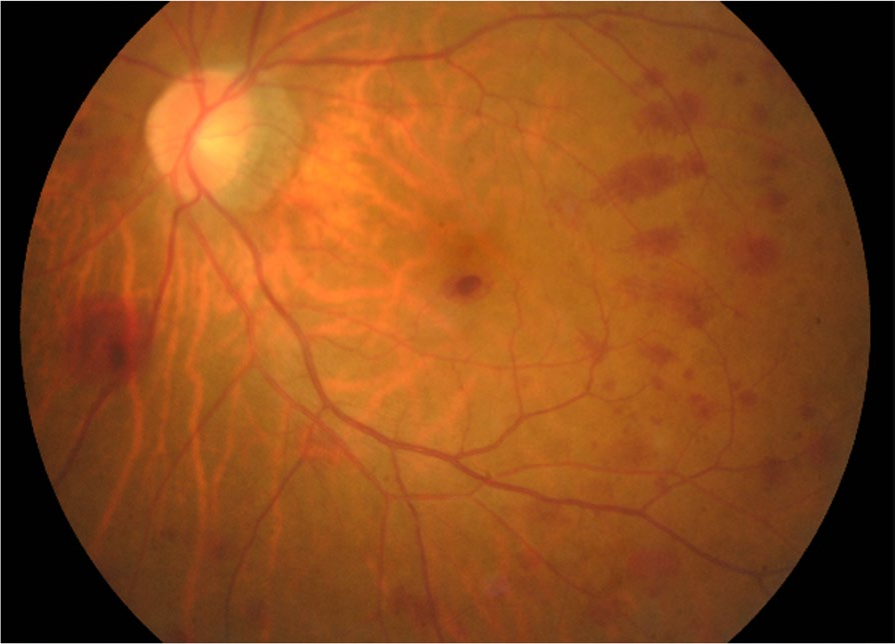
Fundus photograph of the left eye after carotid artery stenting for the left common carotid artery. Examination of the fundus after carotid artery stenting for the left common carotid artery showed diffuse midperipheral retinal hemorrhage

**Fig. 8:**
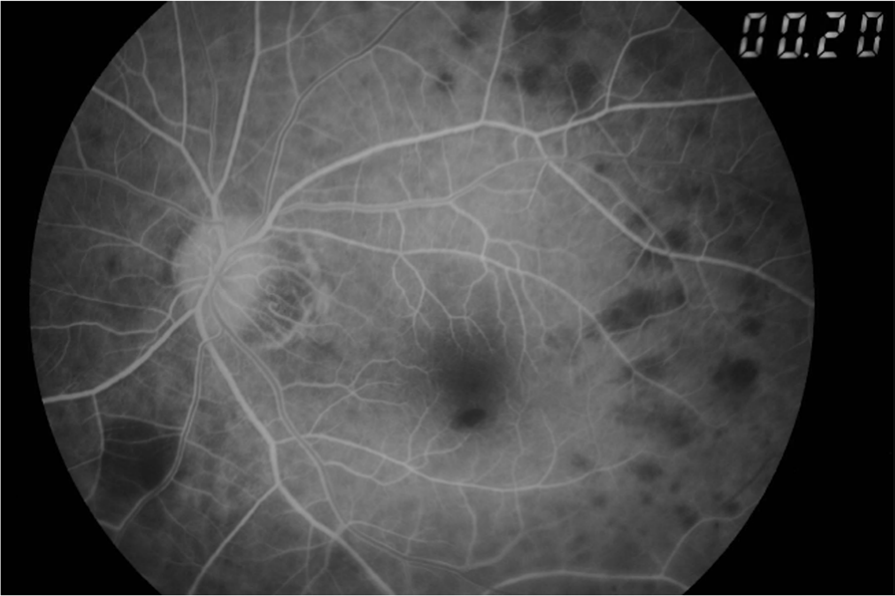
FAG of the left eye after carotid artery stenting for the left common carotid artery. After carotid artery stenting for the left common carotid artery, the corresponding fluorescein angiography image taken at 20 s (arterial phase) demonstrates diffuse blocked fluorescence

On examination, the best corrected visual acuity (BCVA) was counting fingers/20 cm in the right eye and 2/100 in the left eye. The intraocular pressure (IOP) was 21 mmHg in both eyes. No iris rubeosis, inflammation, or relative afferent pupillary defect was noted in either eye. Fundus photograph of the left eye showed venous dilation and attenuated arterioles (Fig. 1). Fluorescein angiography (FAG) image taken at 56 s (arterial phase) demonstrated prolonged arm-to-retina circulation time and a patchy choroidal filling pattern (Fig. 2). Laboratory studies showed erythrocyte sedimentation rate (ESR), 2 mm/hr.; hemoglobin, 14.0 g/dL (mean corpuscular volume, 106.4 /fL); white blood cells (WBC), 22.87 × 103/μL (neutrophils 89%, lymphocytes 8%, monocytes 2%); and platelets 27.0 × 104/μL. Blood chemistry was normal. Chest radiography was unremarkable. Brain magnetic resonance angiography (MRA) showed a total absence of flow in the right internal and external carotid arteries as well as left extracranial carotid arteries (Fig. 3). Ultrasonography of the ophthalmic artery showed reverse blood flow in the bilateral ophthalmic arteries (Fig. 4). Furthermore, aortography revealed total occlusion of bilateral CCA, and computed tomography angiography (CTA) demonstrated nearly total occlusion of the bilateral CCA and nearly total occlusion of the right internal carotid artery (ICA) (Fig. 5 and Fig. 6). Consequently, a tentative diagnosis of OIS with BCCAO was concluded. The patient then was transferred to the cardiology department for further management. After carotid artery stenting for the left common carotid artery, the patient’s BCVA in the left eye improved to 30/100, and examination of the fundus showed diffuse midperipheral retinal hemorrhage (Fig. 7). The corresponding fluorescein angiography image taken at 20 s (arterial phase) demonstrated diffuse blocked fluorescence (Fig. 8).

## Discussion and conclusions

This case demonstrates BCCAO in a patient with primary Sjogren’s syndrome, HTN, and DM. The most common risk factors of CCAO include HTN and a history of smoking [6]. Previous studies also reported that DM is a major risk factor for atherosclerosis which causes carotid artery occlusion [7]. Though this patient denied a smoking history, the possibility of BCCAO due to HTN and DM, in this case, cannot be fully ruled out. However, in patients with HTN and DM, the progression of atherosclerosis should be detectable in MRI [10]. Eccentric wall thickening and the juxtaluminal T2-weighted hyperintensity that is evident in atherosclerotic disease were not seen in our patient. Another reason for HTN and DM may be the minor cause of CCAO in this case is that this patient was a 50-year-old female, both the age and the gender were possibly protective against the ischemic stroke [11].

The radiologic finding of MRA in this patient suggested that nonatherosclerotic causes of the large vessel occlusion, such as inflammatory diseases, were more likely. The possibilities considered were Sjögren’s syndrome and Takayasu arteritis. The age at disease onset, the absence of narrowing of the aorta and/or its primary branches in MRI, and the absence of claudication of the extremities suggest Takayasu arteritis is less likely according to the American College of Rheumatology (ACR) classification criteria [12]. Sjögren’s syndrome is a challenging disorder characterized by several clinical features in different systems, including ocular, cutaneous, and vascular domains [13]. The risk of large-artery involvement in Sjögren’s patient is less often discussed [3]. There are chances that the inflammatory changes due to Sjögren’s syndrome can cause vascular occlusion, but current evidence is not sufficient. This case provides further insight into the complex pathogenesis of the carotid artery involvement in Sjogren’s syndrome, HTN, and DM.

As the ophthalmic artery is a branch of the carotid artery, OIS occurs after the stenosis of the carotid artery [8]. On the other hand, Sjögren’s syndrome can cause systemic inflammation, leading to retinal vasculitis which may also cause visual dysfunction [1]. Therefore, we cannot exclude the possibility that the visual dysfunction in the right eye previously diagnosed as retinal vasculitis was also coincidentally caused by OIS due to BCCAO.

CCAO is an uncommon cause for OIS [9]. The most common clinical features of OIS are rubeosis iridis in the anterior segment and narrowed retinal arteries, dilated and not tortuous retinal veins, retinal hemorrhage, and microaneurysms in the posterior segment [14]. Another characteristic of OIS is ocular or periocular pain due to ocular ischemia [14]. There was no anterior segment sign in this case during the first visit, and the patient also denied ocular pain. Ophthalmologists should be aware that sonography of the ophthalmic artery and carotid artery imaging are important in the differential diagnosis for patients with such atypical presentation.

The management of unilateral CCAO is still debatable because of the low incidence of CCAO [15]. There are several surgical approaches for patients with unilateral CCAO, however, complications from surgery include periprocedural stroke, periprocedural mortality, ipsilateral stroke, restenosis, and re-occlusion [15]. Most patients with unilateral CCAO (68%) received medical treatment instead of surgery [16]. For patients with BCCAO, it is still unknown whether medical or surgical management is optimal due to the limited sample size of previous studies [16].

In summary, we presented a Sjögren’s syndrome patient with OIS due to BCCAO. Large vessel abnormalities should be considered when acute visual loss is found in a patient with Sjögren’s syndrome, HTN, or DM. Various imaging modalities including brain MRA, ultrasonography of the ophthalmic artery, aortography, and CTA are useful for diagnosing diseases with complicated pathogenesis, such as in this case.

## Data Availability

The datasets used and/or analyzed during the current study are available from the corresponding author on reasonable request.

## Abbreviations

BCCAO: Bilateral common carotid artery occlusion
CCAO: Common carotid artery occlusion
CT: Computed tomography
CTA: Computed tomography angiography
DM: diabetes mellitus
HTN: hypertension
ICA: Internal carotid artery
MRA: Magnetic resonance angiography
OIS: Ocular ischemic syndrome
